# Method for building segmentation and extraction from high-resolution remote sensing images based on improved YOLOv5ds

**DOI:** 10.1371/journal.pone.0317106

**Published:** 2025-03-18

**Authors:** Fangzhe Chang, Tianyue Ma, Dantong Wang, Shoujie Zhu, Dengping Li, Shuntian Feng, Xiaoyong Fan

**Affiliations:** 1 Hebei Provincial Communication Planning, Design, and Research Institute Co., Ltd Town Renewal Research Center, Shijiazhuang, China,; 2 School of Geoscience and Surveying Engineering, China University of Mining and Technology (Beijing), Beijing, China,; 3 Hangzhou Survey, Design and Research Institute Co., Ltd, New Smart City Research Center, Hangzhou, China,; 4 Department of Safety Supervision, China MCC22 Group Co., Ltd, Tangshan, China,; 5 China Telecom Digital City Technology Co., Ltd, Intelligent Transport Systems Product Line, Baoding, China; Shijiazhuang Tiedao University, CHINA

## Abstract

To address challenges in remote sensing images, such as the abundance of buildings, difficulty in contour extraction, and slow update speeds, a high-resolution remote sensing image building segmentation and extraction method based on the YOLOv5ds network structure was proposed using Gaofen-2 images. This method, named YOLOv5ds-RC, comprises three primary components: target detection, semantic segmentation, and edge optimization. In the semantic segmentation module, an upsampling and multiple convolutional layers branch out from the second feature fusion layer of the Feature Pyramid Networks (FPN), producing a category mapping image that matches the original image size. For edge optimization, a Raster compression module is incorporated at the end of the segmentation network to refine the segmentation contours. This approach enables effective segmentation of Gaofen-2 images, achieving detailed results at the individual building scale across urban areas and facilitating rapid contour optimization and extraction. Experimental results indicate that YOLOv5ds-RC achieves an accuracy of 0.8849, a recall of 0.63904, an average precision (AP) at 0.5 of 0.75863, and a mean average precision (mAP) from 0.5 to 0.95 of 0.47388. These metrics significantly surpass those of the original YOLOv5ds, which recorded values of 0.81483 for accuracy, 0.51332 for recall, 0.63552 for AP at 0.5, and 0.34922 for mAP. The algorithm effectively corrects target displacement deviations in non-orthogonal images and achieves more objective and accurate contour extraction, meeting the requirements for rapid extraction. Due to these features, YOLOv5ds-RC can further enhance fully automated rapid extraction and historical change analysis in land use change monitoring.

## Introduction

In recent years, advancements in satellite technology, sensor resolution, and neural network image recognition have enabled the integration of high-resolution remote sensing images with advanced deep learning techniques. This combination has shown significant potential for surface feature identification in remote sensing detection [1]. China civilian land observation satellites include the Ziyuan series, the Environment and Disaster Reduction series, the Mapping series, the Gaofen series, the Shijian series, the Electromagnetic series, and the PPP (Public-Private Partnership) satellites. The Gaofen series, notable for its highest resolution reaching sub-meter levels, includes ten satellites, all of which are operational except Gaofen-5 [2]. The data from these satellites are extensively used in various fields, such as land resources, urban planning, environmental monitoring, disaster prevention, agriculture, forestry, water conservancy, meteorology, e-government, statistics, marine studies, mapping, and significant national projects, contributing greatly to social development [3].

Building extraction methods from remote sensing images have evolved into two main categories: 1)Traditional Remote Sensing Interpretation Methods: These involve classification based on various index methods, color, texture, shape, and more [4]; 2) Machine Learning or Deep Learning Methods: These methods leverage the powerful convolutional computing capabilities of computers to extract image features and conduct complex classification. Öztimur Karadağ et al. [5] improved ICT-Net by combining it with the watershed segmentation algorithm for building extraction, which increased the recall rate of building instances by 22.9% compared to the original ICT-Net network. Zhang and Wang [6], used an improved MA-Unet, incorporating an attention module, achieving better accuracy than the original U-Net network. Wang et al. [7] used the DeepLabv3 + network model to establish an automated high-resolution remote sensing image building extraction method. Raghavan et al. [8] proposed an algorithm is proposed which extends the convolutional neural network for pixel-wise classification of images.

Currently, deep learning methods for target recognition are categorized into one-stage and two-stage detection algorithms. One-stage algorithms, including the YOLO series [9], G-CNN [10], SSD [11], and RON [12], perform regression and classification directly without generating candidate boxes. In contrast, two-stage algorithms, such as R-CNN [13], SPP [14], Fast R-CNN [15], Faster R-CNN [16], and Mask R-CNN [17], first generate candidate boxes and then perform regression and classification using convolutional neural networks. Two-stage algorithms generally achieve higher accuracy and precision, making them ideal for detecting precise targets at various scales. One-stage algorithms, known for their speed, are suitable for real-time detection tasks involving video, cameras, and large datasets. Considering factors such as research scale, sample size, and time requirements, this experiment focuses on the one-stage YOLO series model for further research and improvement.

In 2015, Joseph Redmon and others proposed YOLOv1, which has faster detection speed than two-stage algorithms like R-CNN but with slightly lower accuracy. In 2016, Joseph Redmon upgraded the YOLOv1 algorithm to YOLOv2 [18], which improved the number and accuracy of detected categories by jointly training objects and classifications.YOLOv3 [19], another upgrade to the YOLO series by Joseph Redmon, introduced a more complex structure with a pyramid network and classification via entropy loss functions. This version improved algorithm performance, with slightly reduced speed but higher accuracy. In 2020, Alexey Bochkovskiy proposed YOLOv4 [20], incorporating a wide range of functional networks to form an efficient object detection model through continuous optimization and parameter tuning. In June 2020, Glenn Jocher and others released YOLOv5, which is significantly more lightweight than YOLOv4 and improves computing speed while maintaining accuracy. This study further enhances the original YOLOv5ds network, resulting in the YOLOv5ds-RC model. Each model was evaluated using a test set, and their performance in building segmentation and detection was compared. The evaluation metric values were analyzed to determine the effectiveness of the improvements.

## Materials and methods

### Study area and dataset creation

**Fig 1:**
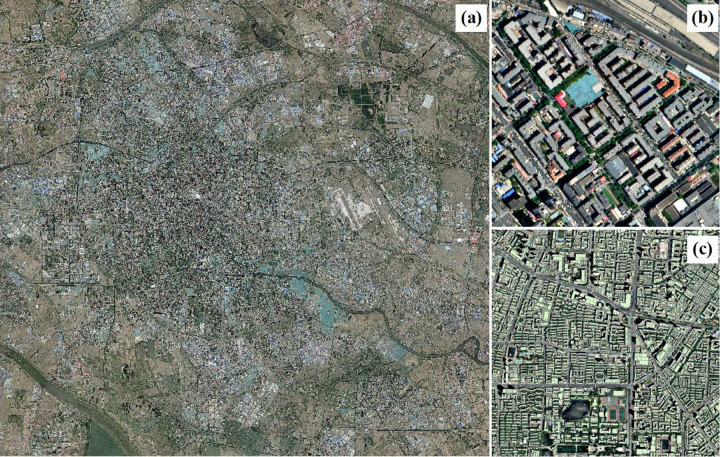
Study area image and datasets. (a) Original image of the experimental area; (b) Partial view of Gaofen-2 image; (c) Annotation data of building base map.

**Fig 2:**
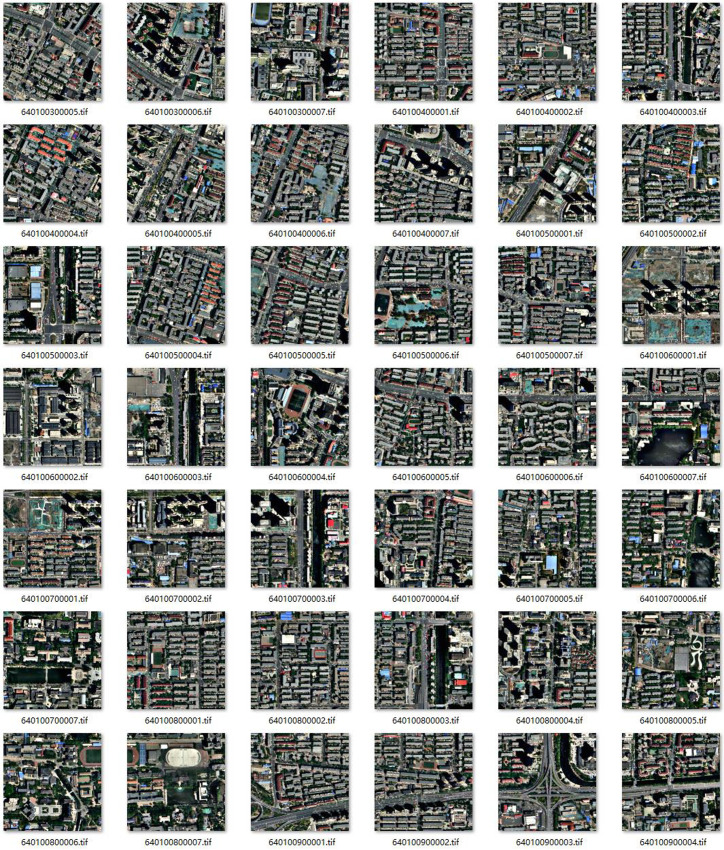
Cropped image block diagram.

**Fig 3:**
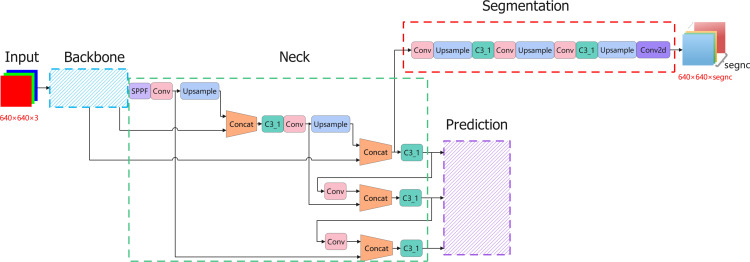
Schematic diagram of YOLOv5ds structure.

The experimental area covers the central urban district of Tianjin, including the city and surrounding regions, spanning 1450 km^2^ (Fig 1a). The data consist of recent Gaofen-2 remote sensing images of the entire city, with a resolution of 0.8 meters (Fig 2b). Sample annotations include historical building base maps of Tianjin, featuring contour vector diagrams of each building over the years (Fig 3c), used for accurate boundary delineation and analysis.

A crucial aspect of sample preparation in this experiment involves annotating buildings with their actual geographic coordinates. Due to deviations between annotations of non-orthographic images and the original building images, this study aims to accurately extract buildings’ true geographic coordinates by training the model with specially annotated samples. Using a custom Python tool, the images were cropped into 640 × 640 resolution blocks and corresponding annotated vector datasets (Fig 2). This process produced 3520 valid image blocks and 3520 valid vector datasets. These datasets were randomly split into training, validation, and test sets in a 7:2:1 ratio, resulting in 2464 training images, 704 validation images, and 352 test images.

### YOLOv5ds structure optimization

YOLOv5ds is an enhanced version of the YOLOv5 framework by Python, designed to simultaneously perform two computer vision tasks: object detection and semantic segmentation. The algorithm designed in this study is an improvement on the newer version of YOLOv5 (v6.1). The network structure of YOLOv5 v6.1 is illustrated in Fig 3.

The five components and corresponding functional structures of YOLOv5ds are as follows:

1. Input: The input images used in this study are 640 × 640-sized 3-channel RGB images. Data augmentation techniques like Mosaic and MixUp are enabled, adaptive anchor box calculation is done using K-means, and adaptive image scaling strategies are applied to preprocess the input images.
2. Backbone: Extracts image features, comprising the CSP (Cross-Stage Partial) structure [21] and SPPF [22] (Spatial Pyramid Pooling) module. The CSP module includes two types of connections: direct convolution operations and an additional skip connection. This helps accelerate network computation and enrich feature extraction. The SPPF module consists of one convolution operation and three identical max-pooling layers (kernel size = 5) connected in series. The four resulting tensors are concatenated along the dimension direction to form a larger receptive field, beneficial for model learning.
3. Neck: Enhances image feature extraction, designed with a combination of FPN [23] and PAN (Path Aggregation Network) structures [24]. The FPN structure addresses the multi-scale problem in object detection by predicting through multi-scale feature fusion. Two PAN structures are integrated into the FPN for convolution and down-sampling operations, ultimately yielding three feature map outputs at different sizes: 1/32, 1/16, and 1/8 of the original image.
4. Prediction Output: Uses CIOU_Loss and CIOU_NMS [25] to predict training results, outputting three feature maps at different scales, each with 3 × (5 +  number of detection classes) channels.
5. Segmentation Network: Gradually restores the image to its original size through a series of convolutions and up-sampling, and calculates multi-class loss using CrossEntropyLoss [26]. The final segmentation feature map output has the same size as the original image, with a number of channels equal to the number of segmentation classes.

**Fig 4:**
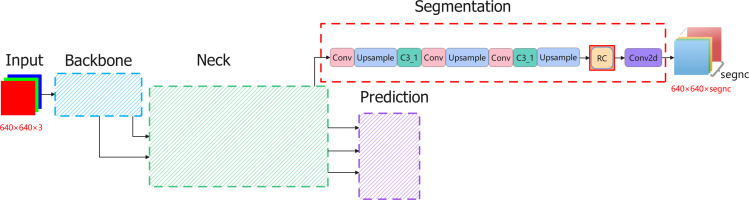
Schematic diagram of YOLOv5ds-RC structure.

An RC module is added to the YOLOv5ds framework, resulting in an improved version called YOLOv5ds-RC (Fig 4). The primary improvement of YOLOv5ds-RC is the addition of an RC pipeline after the Neck. The RC module was excluded from the training process but was incorporated into the Segmentation branch derived from the Neck part of YOLOv5ds. After being processed by the RC module, the output is a segmentation map with smoothed edges. The output from the Neck is processed through the Segmentation and RC workflows to generate the final image.

**Fig 5:**
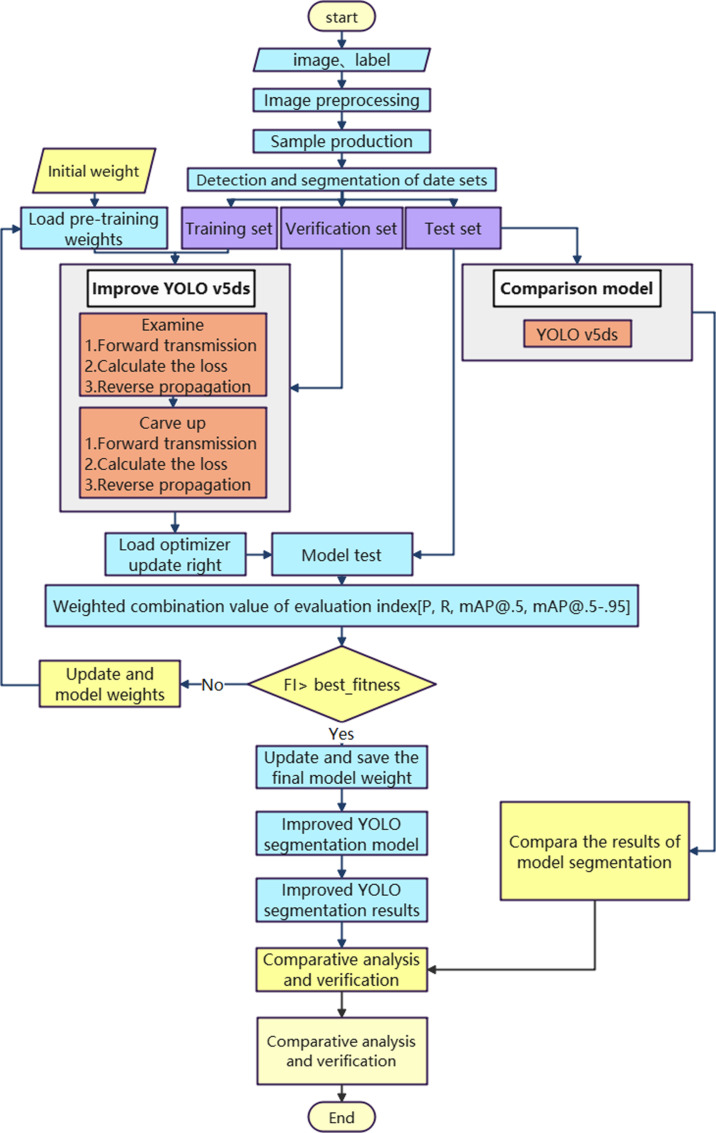
Technical route of high-resolution remote sensing image segmentation and extraction using YOLOv5ds.

The process for building segmentation and extraction in high-resolution remote sensing images, based on the improved YOLOv5ds, involves several steps: creating sample datasets, improving the model, training model weights, testing the model, and comparing the results. The technical workflow is illustrated in Fig 5. The parallelograms represent input data and parameters, while the rectangles represent the processes within nodes. The gray boxes indicate the models used for training and prediction. The flowchart is primarily divided into three sections: the first section deals with the processing of the training set, validation set, and sample set for images and labels; the middle section focuses on the training, evaluation, and iterative learning of various deep learning models; and the final section involves comparing and validating the segmentation results from multiple trained models.

### Raster compression algorithm

**Fig 6:**
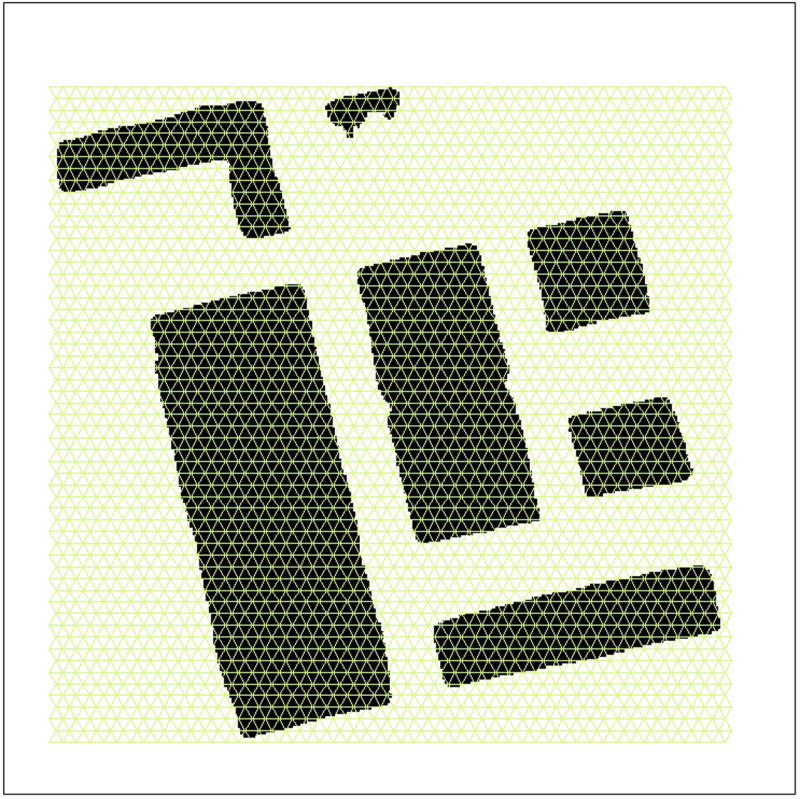
The principle of surface compression.

To obtain more accurate building boundary contours, the Raster Compression (RC) algorithm is integrated into the network structure. The algorithm first generates a grid of small equilateral triangles that covers the entire image (as illustrated in Fig 6, with the triangles enlarged). It then evaluates the contact area between the original building and this grid, identifying a composite polyline that minimizes the sum of deviation integrals for all segments within a specified tolerance range in the same direction. The generated polyline is further tested to ensure: 1. The endpoints of the polyline are within the tolerance range of the original edge. 2. The polyline aligns with the original edge without changing direction.

**Fig 7:**
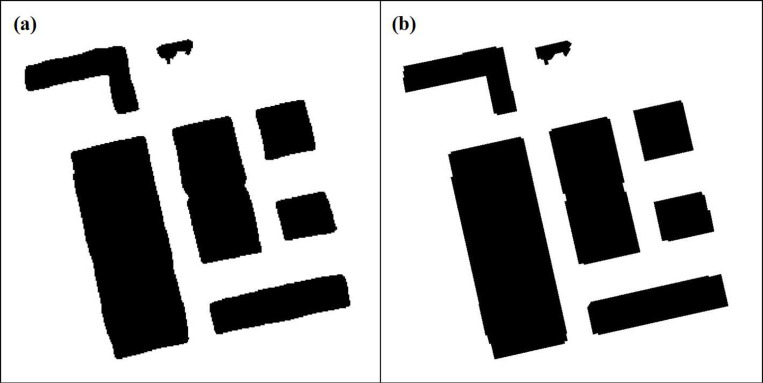
Comparison chart of surface compression. (a) Original raster; (b) Area Compression Corrected Raster.

In the segmentation results produced by the YOLOv5ds algorithm, artifacts are present along the extracted raster edges (Fig 7a). This algorithm processes the raster edges of buildings with long straight or diagonal edges to achieve orthogonal alignment, contour filling, and artifact elimination (Fig 7b).

### Experimental process

The workstation configuration used in the experiment includes a 64-bit Windows 10 operating system, an Intel Xeon W-10885M processor, 128GB of memory, and an NVIDIA Quadro RTX 5000 graphics card. The sample preparation development environment is Python 3.8. The experiment involves training multiple models on the same sample set, calculating specific accuracy metrics, and performing a longitudinal comparison. The experimental parameter configurations for training the YOLOv5ds and YOLOv5ds-RC networks are detailed in Table 1, outlining the key settings and adjustments used to optimize the training process.

**Table 1:** Configuration of training parameters.

Hyperparameter name	Value
Batchsize	4
Epoch	1000
Initial learning rate	0.01
Cyclical learning rate	0.1
Momentum factor	0.937
Weight decay coefficient	0.0005
IOU	0.2

### Accuracy verification

To evaluate the enhancement effects of the proposed algorithm on object detection and semantic segmentation, the following metrics were used: precision, recall, mAP, average inference time, and mean intersection over union (MIoU). Except for average inference time, all values range between 0 and 1. Here, TP represents true positives (correctly identified buildings), and FN represents false negatives (incorrectly identified buildings).

1) Precision: The ratio of true positives to the total predicted positives, as shown in Equation (1).

$$Precision=\frac{TP+FP}{TP}$$ (1)

2) Recall: The ratio of true positives to the total actual positives, as shown in Equation (2).

$$Recall=\frac{TP+FN}{TP}$$ (2)

3) AP: A key metric for evaluating model performance, representing the average precision across different recall points. It is the area under the precision-recall (PR) curve, calculated as shown in Equation (3).

$$AP=\int_{0}^{1}PdR$$ (3)

4) mAP: The mean AP across all categories, calculated as shown in Equation (4).

$$mAP=\frac{\sum_{i=1}^{k}AP_{i}}{k}$$ (4)

mAP@0.5: This metric calculates mAP by setting the intersection over union (IoU) threshold to 0.5. It involves computing AP for each class across all images, and then averaging the APs of all classes to obtain the mAP. mAP@[.5:.95]represents the average mAP calculated over different IoU thresholds, ranging from 0.5 to 0.95 in increments of 0.05 (i.e., 0.5, 0.55, 0.6, 0.65, 0.7, 0.75, 0.8, 0.85, 0.9, and 0.95).

5) Average Inference Time: The mean time required for the model to process all images in the dataset, reflecting its inference speed.
6) MIoU: A standard metric for semantic segmentation, averaging the IoU for each category between the ground truth and predicted segmentation, as shown in Equation (5).

$$MIoU=\frac{1}{k+1}\overset{k}{\underset{i=0}{\sum }}\frac{TP}{FN+FP+TP}$$ (5)

## Results

### Recognition results

**Fig 8:**
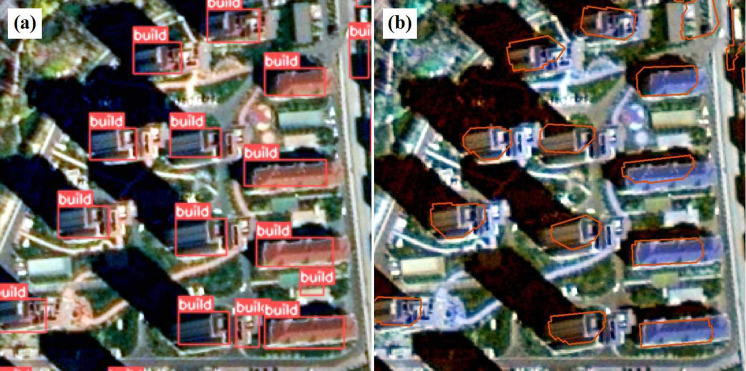
Example of detection and segmentation result process. (a) Example of building non-orthorectification detection result; (b) Example of building non-orthorectification segmentation result.

The model weights derived from the improved YOLOv5ds algorithm are employed for extracting results, including detection and segmentation outcomes (for clarity, the segmentation results have been center-erased). A partial display of these results is shown in Fig 8. According to the building detection results in Fig 8(a), the model effectively addresses the challenge of obtaining the actual coordinates of buildings in non-orthographic images. Given that the resolution of high-resolution images is 0.8m, which is considerably lower than that of UAV images, accurately extracting building contours remains a challenging aspect of this experiment. The building segmentation results in Fig 8(b) illustrate that the YOLOv5ds-RC model not only accurately extracts the actual coordinates of buildings but also significantly enhances building contours through improvements in the Raster Compression algorithm.

### Recognition result and accuracy comparative analysis

**Fig 9:**
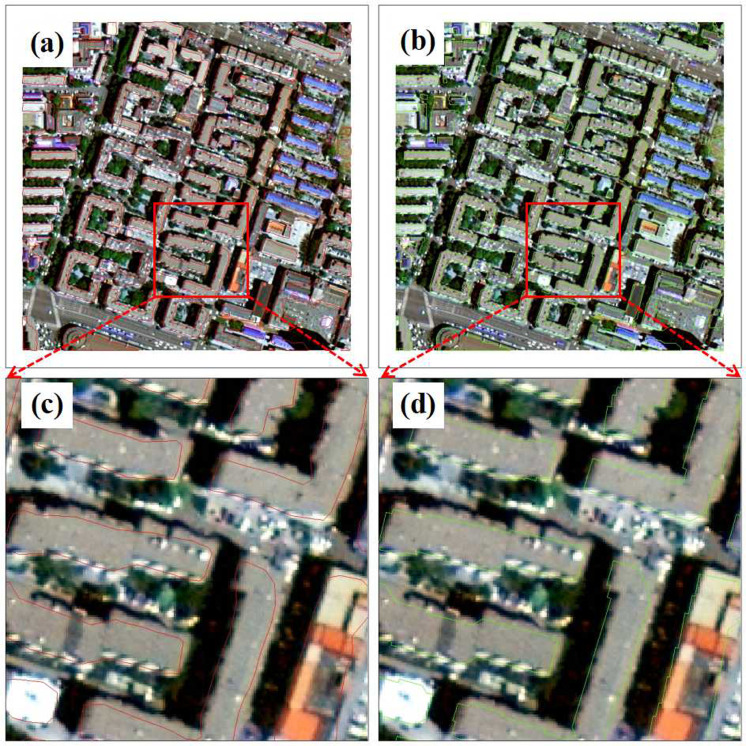
Comparison diagram of improvement. (a) The recognition results of the test image block before the improvement of YOLOv5ds; (b) The recognition results of the test image block of YOLOv5ds-RC; (c) Local magnification and comparison of recognition results before improvement; (d) Local magnification and recognition results comparison of YOLOv5ds-RC.

Fig 9 shows the recognition performance of the original YOLOv5ds and the improved YOLOv5ds-RC on the test set. According to the comparison between Fig 9(a) and Fig 9(b), both model detection results generally meet the requirements. The local comparison between the extraction results of the two models in Fig 9(c) and Fig 9(d) shows that the contours extracted by YOLOv5ds-RC are more objective and reasonable, conforming to building characteristics.

Table 2 compares the accuracy performance of the original YOLOv5ds and the improved YOLOv5ds-RC on the test set. Both models achieved good detection results in building extraction. Based on the comparison of extraction effects, it has been proven that YOLOv5ds-RC’s segmentation performance is superior to that of YOLOv5ds. The AP values in Table 2 demonstrate the same result from an evaluation metric perspective.

**Table 2:** Accuracy comparison results.

Model	P	R	AP:0.5	mAP: (0.5:0.95)	Average inference time (ms)	MIoU
YOLOv5ds	0.81483	0.51332	0.63552	0.34922	5.0	0.71564
YOLOv5ds-RC	0.8849	0.63904	0.75863	0.47388	5.7	0.79802

### YOLOv5ds-RC application analysis

**Fig 10:**
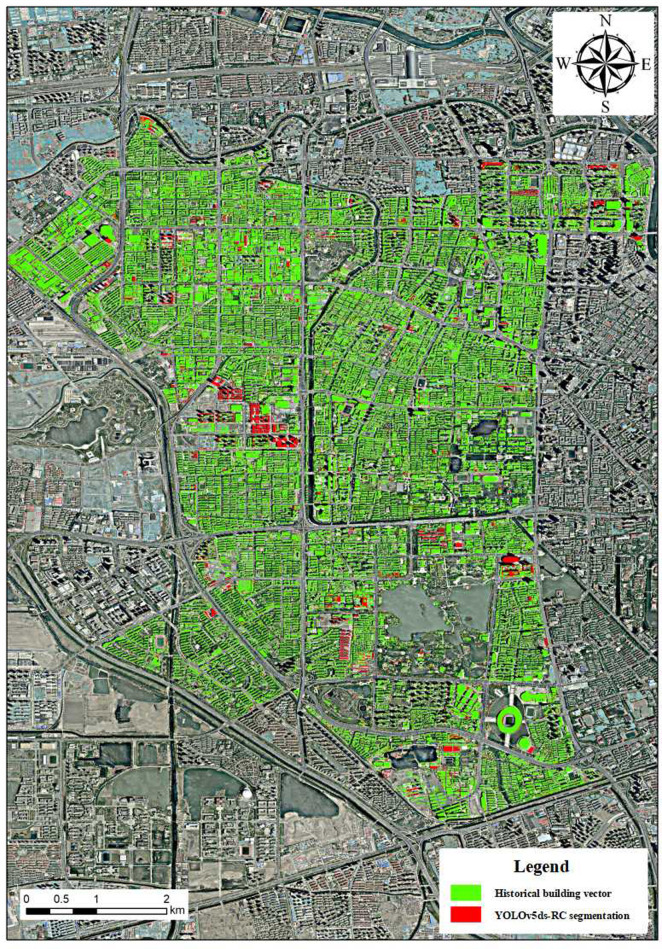
Comparison of architectural history vector and remote sensing segmentation results.

The YOLOv5ds-RC algorithm was applied to high-resolution remote sensing images of the study area for segmentation. Green indicates historical building vector data from earlier in the same year, while red shows the remote sensing segmentation results after manual correction and verification (Fig 10). Analyzing the red areas in different parts of the figure reveals that the central western part has the most dense and largest red area, while other regions show varying degrees of red segmentation. This indicates changes in building distribution.

## Discussion and limitations

In this study, we used an improved version of YOLOv5ds—a deep learning framework incorporating an RC module. Traditional image recognition methods, such as feature extraction techniques based on SIFT and SURF, along with machine learning algorithms like SVM, perform well in simple scenarios. However, they often require additional preprocessing steps in complex environments and struggle with real-time performance. In contrast, the YOLO algorithm is highly efficient in recognizing buildings within complex urban settings, offering superior adaptability. Our findings are consistent with prior research. Compared to other deep learning approaches like ResNet and Faster R-CNN, YOLO demonstrates a significant advantage in real-time performance. While YOLO may slightly underperform in detecting small objects or in crowded scenes compared to methods like Faster R-CNN, its overall accuracy and speed in building detection make it the preferred choice for many practical applications. For example, YOLO is able to maintain high detection accuracy while significantly reducing processing time in building detection from drone and satellite imagery. Studies have explored the application of the YOLO algorithm in drone imagery, showing that it strikes an excellent balance between accuracy and detection speed, making it well-suited for scenarios requiring high real-time performance [27]. Overall, the YOLO algorithm, due to its efficiency and accuracy, holds broad application potential in building recognition tasks. However, for more complex scenarios and multi-object detection tasks, future improvements may benefit from integrating advanced technologies, such as attention mechanisms and Transformer models, to further enhance performance.

**Fig 11:**
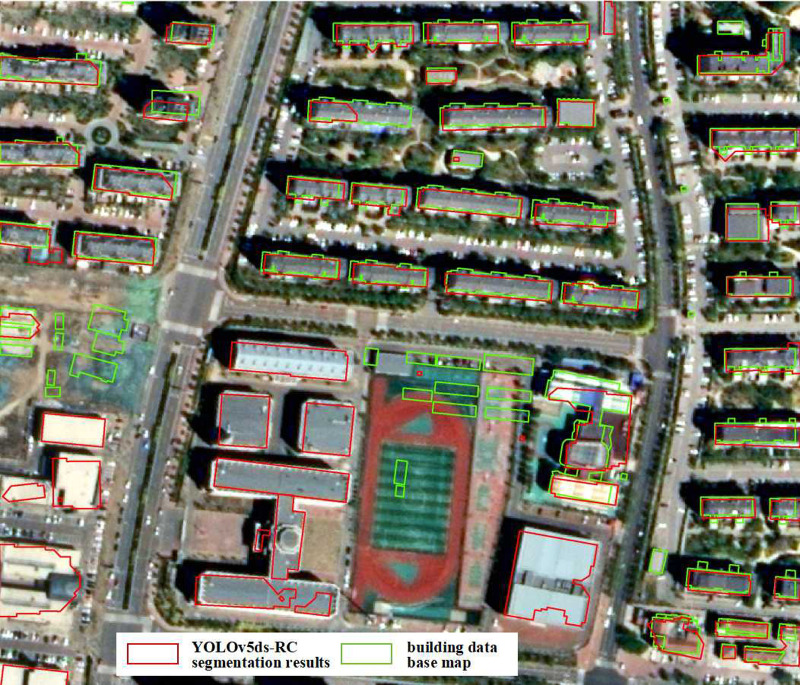
Application diagram of automatic monitoring of land use change.

The comparison of YOLOv5ds-RC’s accuracy demonstrates its superiority, and the experiment further validates its effectiveness through specific applications. These applications include: 1. Monitoring land-use changes; 2. Updating and correcting building data base maps. The experiment extracted buildings from the test image set using YOLOv5ds-RC and ultimately merged the results into a panoramic building contour map. Fig 11 shows the comparison results of a specific image block during extraction, where the green vectors represent the building data base map, and the red data represent the segmentation results of the YOLOv5ds-RC model. From the comparison of the two results, it can be concluded that, on the one hand, the building base map is outdated. For instance, some buildings in the middle-left of F Georeferenig. 11 have disappeared or been converted to other types of land cover, and new buildings have appeared in the lower-left that are not reflected in the green vectors. On the other hand, because the accuracy of YOLOv5ds-RC reaches 0.88 and the mAP reaches 0.47, almost all buildings can be successfully detected, while ensuring the accuracy of extracting most building contours.

The extraction results of YOLOv5ds-RC can supplement and correct existing building base maps, providing a fully automated and rapid method for detecting illegal constructions. Furthermore, identifying buildings in historical images can serve as evidence to determine whether a building was constructed illegally.

Currently, the training accuracy of the model is limited by factors affecting sample quality, including the number and accuracy of labels, image resolution, and capture time. The extraction results exhibit some instances of missed and incorrect detections, and the boundary contour results still differ from manual extraction. In future research, we will continue to improve sample quality, refine the model methodology, and enhance recognition accuracy.

## Conclusion

YOLOv5ds-RC demonstrates superior building extraction accuracy compared to YOLOv5ds, with the AP: 0.5 metric showing a significant improvement of 12%. Non-orthographic imaging leads to image displacement at building tops; however, YOLOv5ds-RC successfully extracts the true geographic contours of buildings from Tianjin’s high-resolution remote sensing images. This method enables automated rapid extraction and historical change analysis for effective monitoring of land-use changes.
